# Are Regional Habitat Models Useful at a Local-Scale? A Case Study of Threatened and Common Insectivorous Bats in South-Eastern Australia

**DOI:** 10.1371/journal.pone.0072420

**Published:** 2013-08-19

**Authors:** Anna McConville, Bradley S. Law, Michael J. Mahony

**Affiliations:** 1 School of Environmental and Life Sciences, University of Newcastle, Callaghan, New South Wales, Australia; 2 Forest Science Centre, Department of Primary Industries, Beecroft, New South Wales, Australia; Università degli Studi di Napoli Federico II, Italy

## Abstract

Habitat modelling and predictive mapping are important tools for conservation planning, particularly for lesser known species such as many insectivorous bats. However, the scale at which modelling is undertaken can affect the predictive accuracy and restrict the use of the model at different scales. We assessed the validity of existing regional-scale habitat models at a local-scale and contrasted the habitat use of two morphologically similar species with differing conservation status (*Mormopterus norfolkensis* and *Mormopterus* species 2). We used negative binomial generalised linear models created from indices of activity and environmental variables collected from systematic acoustic surveys. We found that habitat type (based on vegetation community) best explained activity of both species, which were more active in floodplain areas, with most foraging activity recorded in the freshwater wetland habitat type. The threatened *M. norfolkensis* avoided urban areas, which contrasts with *M*. species 2 which occurred frequently in urban bushland. We found that the broad habitat types predicted from local-scale models were generally consistent with those from regional-scale models. However, threshold-dependent accuracy measures indicated a poor fit and we advise caution be applied when using the regional models at a fine scale, particularly when the consequences of false negatives or positives are severe. Additionally, our study illustrates that habitat type classifications can be important predictors and we suggest they are more practical for conservation than complex combinations of raw variables, as they are easily communicated to land managers.

## Introduction

Habitat models [1], [2] and predictive mapping [3], [4] have been successful at classifying habitat of wide-ranging or lesser known species. Providing such models are validated [5], predictive maps can be used to direct conservation effort, identify areas for survey and research, and investigate disturbance effects [6]–[10]. Habitat selection operates at many spatial scales [11] and habitat modelling studies should select a scale that is appropriate to both the species and the model’s intended use [12]. Habitat models prepared across the entire distribution of a species are likely to describe only broad patterns, whilst models based on a single population or family group are likely to contain location-specific variables that are unable to be extrapolated to broader scales [13]. Both broad- and fine-scale habitat models have value. For example, landscape-scale studies are useful to identify trends in habitat use by functional groups, such as the use of urban areas by insectivorous bats [14]–[16]. Additionally, studies that are undertaken at a fine-scale may identify factors that are important for conservation management that were unable to be identified from broader studies [17], [18]. For land managers that may use habitat model predictions for land-use planning, it is essential to know whether models may be accurately applied to other spatial scales.

The factors that influence habitat use by a species are poorly understood for many species of conservation concern. Yet this information is fundamental to understanding threatening processes and to the development of adequate conservation strategies. *Mormopterus norfolkensis* Gray, 1839 is a hollow-roosting insectivorous bat species of which little is known. It occurs on the east coast of Australia and is listed as vulnerable under the New South Wales (NSW) *Threatened Species Conservation Act 1995* and as vulnerable C1 under the International Union for Conservation of Nature red list [19]. A morphologically similar species, *Mormopterus* species 2 [20], occurs in sympatry with *M. norfolkensis* in some parts of its range. Both species have morphological characteristics (high aspect ratio and high wing loading) and echolocation call designs (low frequency) indicative of species’ that are adapted to foraging in open habitats [21]–[24]. Regional-scale habitat models based on systematically collected presence/absence data have been previously developed for both species (hereafter referred to as the ‘regional-scale models’) and indicate differences between the species in relation to the use of urban and floodplain habitats [25]. However, in a similar manner to the effect of false negatives on habitat models [26], it is possible that models created using presence/absence data inflate the importance of (and possibly over-predict) habitat for highly mobile species by assigning the same value to a site which had continuous foraging activity as a site where only a single animal passed by (a false positive).

The aim of this study was to determine if local-scale habitat associations, based on an index of habitat use (activity levels), are consistent with the predictions of regional-scale habitat models that were developed using presence/absence data. Additionally, we aimed to explore whether morphologically similar and sympatric species use different habitats by contrasting habitat models of *M. norfolkensis* with those of *M*. species 2. This was achieved with systematic local acoustic surveys in a variety of habitats, centred on a large roosting population of *M. norfolkensis* and nested within the extent of the previous regional-scale habitat modelling. We calculated different accuracy measures to explore model fit and predicted that if habitat use at a local-scale is consistent with regional-scale models [25], both species would be more likely to use floodplain habitats and *M. norfolkensis* would be less likely to occur in urban areas.

## Methods

### Study area

**Figure 1:**
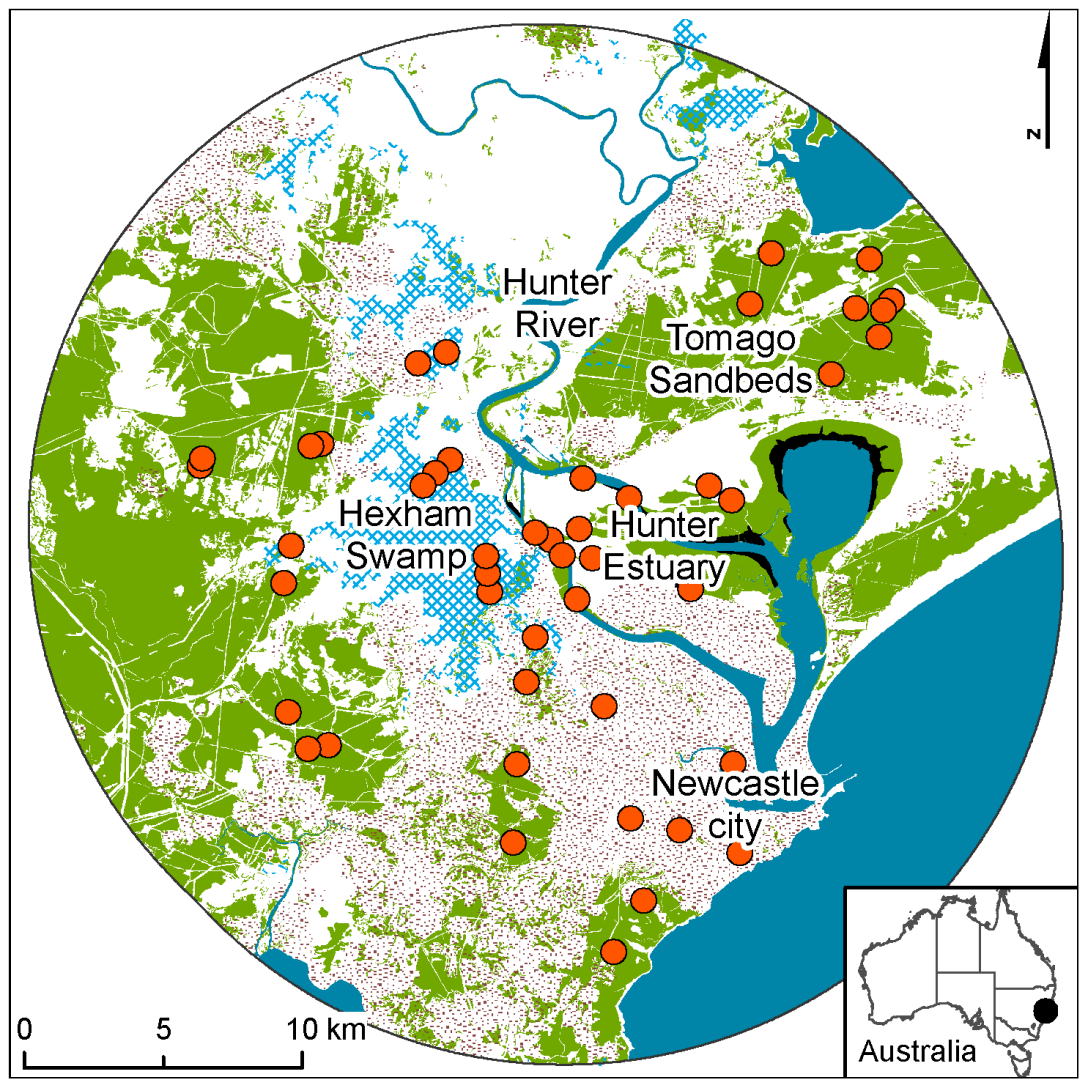
Study area location. Sample sites (orange circle), woody vegetation cover (green shading), mature mangrove forest (black shading), freshwater wetland (blue cross hatch), urban land-use (brown stipple) and major water bodies (blue shading) within 15 km.

The Hunter Estuary enters the Pacific Ocean at the Port of Newcastle (32°55’36”S 151°46’44”E) on the east coast of Australia (Figure 1). The area experiences a warm temperate climate (average monthly temperatures 8.4 – 25.6 °C) and average annual rainfall of 1134 mm [27]. The Hunter Estuary contains high quality estuarine vegetation communities, with the second largest area of mangroves (1600 ha) and the third largest area of saltmarsh (600 ha) in NSW [28]. Areas surrounding the Hunter Estuary have been highly modified by a long history of agriculture, coal mining and port-associated industries. The fertile floodplain of the Hunter River has been extensively cleared for agriculture with only small patches of native vegetation remaining and the construction of flood levies has altered the pattern of river flooding. Hexham Swamp, a large 2500 ha wetland dominated by freshwater vegetation (since the study, floodgates have been opened to re-establish tidal flushing and more estuarine conditions) and generally lacking trees, occurs in the west of the study area. Further west of Hexham Swamp the topography comprises low undulating hills with moderately nutrient-poor soils [29] that support remnant dry sclerophyll forest vegetation (Figure 1), which is typically young with few hollow-bearing trees (A. McConville pers. obs.). The most intact native vegetation (low open forest, woodland and heath) occurs to the north of the estuary on the flat, sandy and nutrient-poor soils of the Tomago sandbeds, which overlay a groundwater resource that is used locally. This area has been subject to disturbances such as sand mining and construction and the operation of military facilities and other infrastructure.

### Study design and site selection

We used a 15 km buffer around two patches of mangroves in the Hunter Estuary that contained multiple maternity roosts and where over 700 *M. norfolkensis* have been trapped [30], to delineate the study area (approximately 76,500 ha; Figure 1), as the maximum recorded movement of *M. norfolkensis* over one night is 10 km (A. McConville, unpublished data 2012). We centred the study area on a large roosting population of *M. norfolkensis* to focus the study at a local-scale where we knew the species was active. This also assisted with obtaining sufficiently high activity levels to use as a response variable in statistical analyses. We classified the study area into ‘habitat type’ categories based on vegetation communities and human land-use. Habitat type categories were: freshwater wetland, dry sclerophyll forest, dry sclerophyll forest on sand; urban bushland; urban matrix; mature mangrove forest; and swamp oak forest. Small, distinct habitat types (e.g. coastal heath) and those that merged with other communities (e.g. saltmarsh) were not sampled. The freshwater wetland category included reed and rush vegetation that lacked tree cover and were dominated by *Typha bonariensis* or *Phragmites australis* with some open water. The dry sclerophyll forest habitat type consisted of open forest and woodland vegetation that was usually over 20 m in height, occurred on undulating slopes and was dominated by *Corymbia maculata* (spotted gum) and *Eucalyptus fibrosa* (broad-leaved ironbark) with *Eucalyptus punctata* (grey gum) occurring occasionally. The dry sclerophyll forest on sand habitat type was located on mostly flat, sandy plains and consisted of low (10 – 20 m height) open forest and woodland vegetation communities dominated by on flat terrain by *Eucalyptus haemastoma* (scribbly gum) and *Angphophora costata* (smooth-barked apple) or *Eucalyptus parramattensis* subsp. *decadens* (Earp’s gum) and by *Eucalyptus pilularis* (blackbutt) on sandy rises. Urban bushland consisted of remnants (8 – 80 ha) of dry sclerophyll forest that were completely surrounded by the matrix of suburban and industrial development. Urban matrix habitat was located in backyards in well-established suburbs of Newcastle and contained scattered ornamental trees and shrubs. Mangrove forest was mostly a monoculture of *Avicennia marina* subsp. *australasica* (grey mangrove) and the swamp oak forest habitat type consisted of small remnant patches (0.4 – 5 ha) dominated by *Casuarina glauca* (swamp oak). A total of 47 sites were sampled, consisting of nine freshwater wetland, eight dry sclerophyll forest, eight dry sclerophyll forest on sand; five swamp oak forest, five urban bushland, five urban matrix, and seven mangrove forest habitat type sites. Sites were not selected randomly, but rather to achieve a spread of sites within the available habitat type and across the study area. Sites were separated by at least 250 m and were located on both private and public land.

### Bat call sampling

Sampling was undertaken between25 January and 25 February 2011, following the maternity season (after weaning, when young are flying). One bat detector (Anabat II with CFZCAIM or Anabat SD1, Titley Electronics, Balina, Australia) was placed at each sample site to passively record for two entire nights. Up to seven sites were sampled on any one night and 10 different detectors were used during the study. Microphones were housed in a 1 m tall plastic pipe to provide some weather protection and were aimed up at a 45 ° angle. Detectors were set in the open, or aimed along flyways and away from vegetation in forested areas to minimise sound attenuation. Surveys were not conducted on windy or rainy nights or on a full moon to avoid any possible effect of these factors on bat activity [31]. Bat detectors were calibrated prior to sampling to ensure that detector sensitivity was similar across all detectors. The average minimum nightly temperature (sourced from the nearest weather station) was calculated for each site and included as candidate variables in analyses. Rainfall and wind speed data were also collected from nearby weather stations and were used to verify that substantial rainfall or high winds were not experienced during sampling.

We developed a filter in AnalookW [32] to reduce the number of call sequences for analysis by excluding most non-*Mormopterus* calls. The following filter parameters were used: smoothness  =  30; body over 1000 ms; F_c_ = 22–39 kHz; S_c_ = –15–20 OPS; and D_c_ = 3.5 – 20 ms. We tested the performance of the filter on a reference library of *M. norfolkensis*, *M*. species 2 and *M*. species 4 calls (n = 104) and found that 95.4% were correctly identified. The reference calls that were excluded using the filter were reviewed and assessed to be poor quality recordings, mostly from old tape units. To test the filter performance on remotely recorded data, we then ran the filter through manually identified calls from one of the survey sites. In this test, the filter correctly identified 99.4% of calls (n = 639) as *Mormopterus* spp. and we considered this to be an acceptably low level of misclassification.

Bat calls that passed the *Mormopterus* filter were then manually identified using AnalookW to view calls and extract parameters. A key to the identification of *Mormopterus* spp. in the study area was developed based on a broader NSW guide [33] to make the process less subjective. Each pass (call sequence) was assigned to one of three categories - definite, probable or unidentified, according to the confidence with which an identification could be made [34]. During manual identification, feeding buzzes were identified by their characteristic shape [33]. Passes that did not contain any search phase pulses or contained fewer than three pulses were not considered. Definite and probable identifications were included in the analyses. We used total activity (number of passes) recorded for each species, at each site (n = 2 nights), to create habitat models. Additionally, we calculated average nightly bat activity (number of passes per night) to allow comparison with other studies and calculated foraging activity (percentage of passes that contained a feeding buzz) for each *Mormopterus* spp. at each site.

### Landscape variables

We used ArcGIS (version 9.3, ESRI, Redlands, CA, USA) to derive a number of environmental variables describing the sample sites and surrounding area. The slope and elevation layers were derived from a 25 m digital elevation model (DEM), which was interpolated (drainage enforced) using 10 m contours and drainage lines and regions from 1:25,000 topographic maps. We created a vegetation coverage layer from regional vegetation mapping [35] with non-woody vegetation types removed (ie grassland and reeds). We also created a freshwater wetland layer consisting of communities dominated by reeds (both in a natural state and used for cattle grazing) as previous predictive mapping indicated that freshwater wetlands were suitable (typically > 0.5 probability of occurrence [25]). We excluded forested wetland types as they occupied only a relatively small portion of the study area. The urban land-use layer was based on land-use mapping (NSW Landuse) obtained from the NSW Office of Environment and Heritage and the soil type was based broadly on soil landscape mapping [29]. The line-work of all layers was revised following a review of recent aerial photography (ESRI, Redlands, CA, USA, 2001) and to ensure that all layers were consistent. We used ArcGIS with the Patch Analyst extension (version 4.0 [36]) to create a series of patch size and configuration variables from the vegetation dataset (Table S1) broadly following McGarigal and McComb [37]. A patch was considered to be > 10 trees together with a minimum area of 500 m^2^. Core areas were considered to be > 100 m from the vegetation edge.

To determine the spatial scale at which habitat use was occurring, we created a series of concentric buffers at 250 m, 500 m, 1 km, 2.5 km and 5 km around each sample site. We then quantified the amount and configuration of environmental variables (Table S1) within each buffer for each site. Many of the environmental variables calculated from buffers had highly skewed distributions that could not be transformed to a normal distribution, so we converted these to ecologically relevant categories for use in the analyses (Table S1).

### Statistical analyses

We used R [38] to prepare models and undertake other statistical analyses using the MASS [39], PresenceAbsence [40], verification [41] and visreg [42] packages. We use generalised linear models (GLMs) with a negative binomial distribution (glm.nb from MASS package) to model the activity of *M. norfolkensis* and *M*. species 2 against environmental variables (Table S1). We excluded the mangrove forest sites from statistical analyses as the very high activity levels were overly influential, most likely due to the use of mangroves as roosts [30]. We first constructed univariate models for each explanatory variable and species. We then ranked the univariate models by Akaike Information Criterion value corrected for small sample size (AICc) [43], with the highest ranking model having the lowest AICc. To reduce the number of variables offered to final candidate models, the variables within the four highest ranking univariate models were then selected for each species for consideration in the final candidate models. We avoided the use of correlated variables (Pearson’s r > 0.6 or r < –0.6) in final candidate models by removing the variable which was the most difficult to interpret. Where landscape buffer variables of the same category were high ranking, only the highest-ranking buffer value was selected for consideration in the final models [44].The final model set contained all combinations of the four highest-ranking and non-correlated variables for each species. The habitat type variable was not combined with other variables to minimise the potential for model overparamaterisation.

We then calculated AICc and model weight (which is interpreted as the likelihood that the model is the best among the 95% confidence model set) [44] for each of the final candidate models. The best-fitting model for each species was considered to have the lowest AICc ranking and any model within two AICc points of the top model was considered to have strong support [44]. The variation explained by each model was assessed by calculating the Nagelkerke’s R^2^ value [45]. Additionally, to assess the predictive capacity of the models we calculated the area under the curve (AUC) for the receiver operating characteristic (ROC) [46], [47] from the presence/absence records of each species. An AUC value of 0.5 suggests a completely random model and 1 indicates perfect discrimination. The traditional academic point system [48] was used as a rough guide for classifying the fit of each model with AUC values [49] where AUC values under 0.7 are considered poor, values of 0.7 to 0.8 are rated as fair, 0.8 to 0.9 as good and those over 0.9 as excellent. Model residuals were checked for spatial autocorrelation using Moran’s I [50]. We plotted partial response curves for the best-fitting models using the conditional response option in the visreg package [42]. We used a chi-square goodness of fit test to compare percentage foraging activity between habitat types and we excluded habitat types with < 2 feeding buzzes from this analysis (urban matrix and urban bushland for *M. norfolkensis* and urban matrix, urban bushland and swamp oak forest for *M*. species 2). All results are reported as mean ± standard error.

### Validation of regional-scale habitat models

We also used data collected during this study to assess the predictive ability of previous regional-scale habitat mapping undertaken for *M. norfolkensis* and *M*. species 2 within the study area [25]. The regional-scale habitat models were prepared from data collected using bat detectors set in a similar manner as this study (mostly for two nights and aimed along flyways and tracks), within an overlapping study area of 770,000 ha [25]. The regional predictive maps were created from the best-fitting generalised linear mixed models of presence/absence data [25]. See [25] for full methodology used to create regional habitat models.

We used the AUC for the ROC of regional-scale predictions against the local-scale dataset as a threshold-independent measure of predictive accuracy. To do this, we extracted the probability of occurrence value from the regional-scale predictive mapping [25] for each of our sample sites in ArcGIS and converted the activity levels to presence/absence records. Additionally, we used threshold-dependent confusion matrices to calculate the following accuracy measures to describe model predictive ability: sensitivity, specificity, prevalence, correct classification rate (CCR), kappa and true skill statistic [46], [51]. We calculated the occurrence threshold for the confusion matrices as the probability of occurrence value from the regional-scale mapping where sensitivity was equal to specificity for the local-scale data. The kappa and TSS accuracy measures range from − 1 to +1, where a value of 1 indicates perfect agreement and values of zero or less indicate a performance no better than random [51], [52]. Kappa values of 0 – 0.4 are considered to indicate slight to fair model performance, values of 0.4 – 0.6 moderate, 0.6 – 0.8 substantial and 0.8 – 1.0 almost perfect [53] and this ranking was also applied to TSS values.

### Ethics statement

This project was conducted under scientific licence (S132C under National Parks Act 1974; S12460) and as we used only indirect survey methods (ultrasonic detectors to record bat calls) we did not require animal ethics approval.

## Results

**Figure 2:**
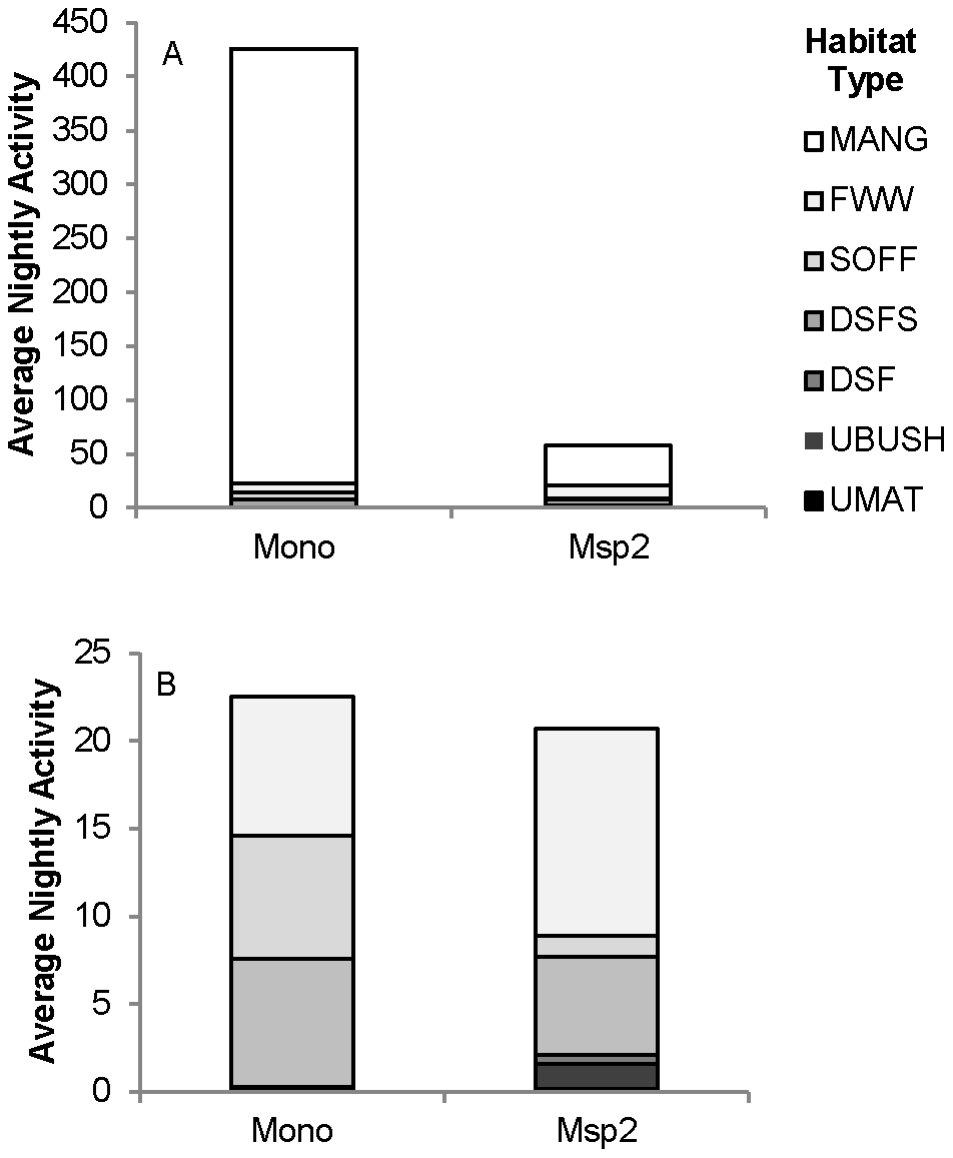
Average nightly bat activity. Average nightly bat activity (number of passes) recorded for each species in the study area across a) all seven landscape categories and b) landscape categories excluding mangrove forest. Abbreviations are: *Mormopterus norfolkensis* (Mono); *M.* species 2 (Msp2); FWW - freshwater wetland; DSF - dry sclerophyll forest and woodland; DSFS - dry sclerophyll forest and woodland on sand; UBUSH - urban bushland remnant; UMAT - urban matrix; MAN - mangrove forest; and SOFF - swamp oak forest.

In all, 5981 *M. norfolkensis* and 854 *M*. species 2 passes were recorded, including 402 *M. norfolkensis* (6.7% of total calls) and 58 *M*. species 2 feeding buzzes (6.8% of total calls) at 47 sites during the study. Site activity levels (63.6±31.7 average nightly passes for *M. norfolkensis* and 9.1±3.3 average nightly passes for *M*. species 2) ranged from 0 – 1272.5 average nightly passes for *M. norfolkensis* and 0 – 144.5 average nightly passes for *M*. species 2. Lower foraging activity was recorded for *M*. species 2 (0.31±0.11, range 0 – 4 feeding buzzes) than *M. norfolkensis* (2.14±1.31, range 0 – 60 feeding buzzes). *Mormopterus* species 2 was more widespread, occurring at 72.3% of sites compared to *M. norfolkensis*, which occurred at 57.4% of sites. *Mormopterus* species 2 was recorded at each of the different habitat types sampled and *M. norfolkensis* was recorded at all habitat types except for the urban matrix (Figure 2). We recorded very high levels of activity of *M. norfolkensis* at mangrove forest sites (403.4±170.7 average nightly passes) being over 50 times greater on average than other habitat types (freshwater wetland had the second highest activity with 7.9±2.1 average nightly passes; Figure 2).

**Figure 3:**
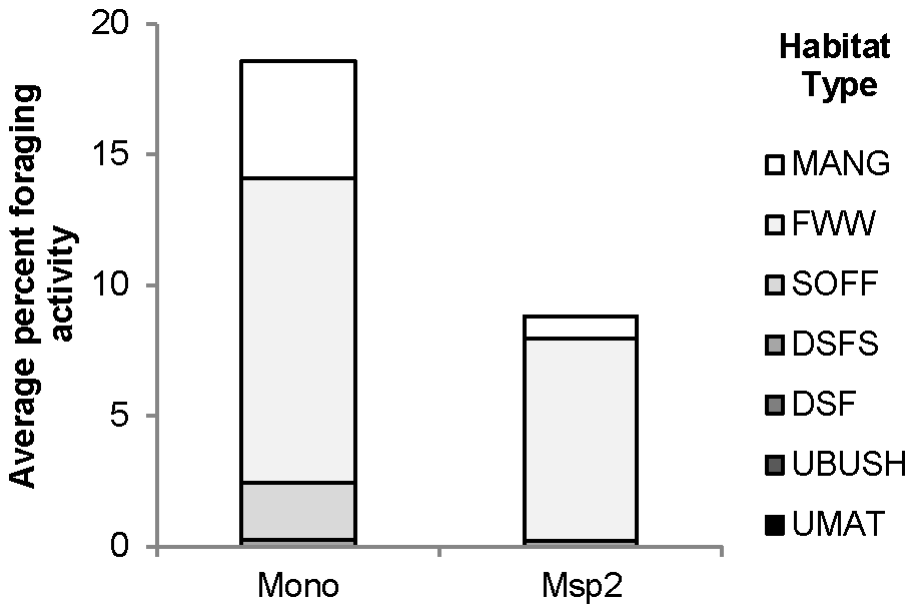
Average percentage foraging activity. Average percentage foraging activity recorded for *Mormopterus norfolkensis* (Mono) and *M.* species 2 (Msp2) across seven different habitat types. FWW - freshwater wetland; DSF - dry sclerophyll forest and woodland; DSFS - dry sclerophyll forest and woodland on sand; UBUSH - urban bushland remnant; UMAT - urban matrix; MAN - mangrove forest; and SOFF - swamp oak forest.

Percentage foraging activity differed significantly between freshwater wetland, mangrove forest, swamp oak forest and dry sclerophyll forest on sand habitat types for *M. norfolkensis* (df = 3, *χ*2 = 25.653, P < 0.001) and also between freshwater wetland, mangrove forest, and dry sclerophyll forest on sand habitats for *M*. species 2 (df = 2, *χ*2 = 33.868, P < 0.001). The freshwater wetland habitat type had the greatest percentage foraging activity for both species (Figure 3). No foraging activity by either species was recorded in the urban habitat types and no *M*. species 2 foraging activity was recorded in swamp oak forest.

### Local-scale habitat models

**Figure 4:**
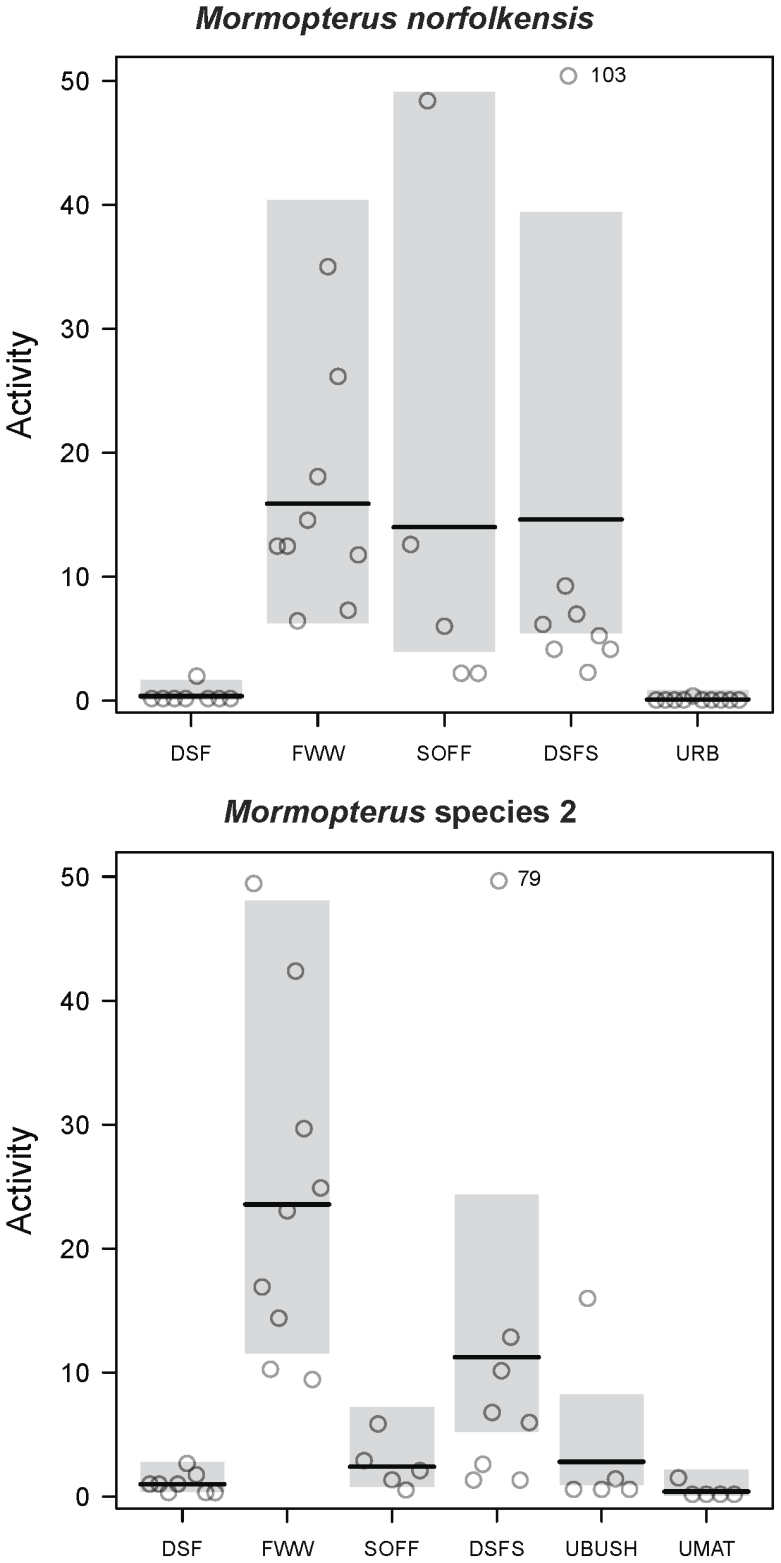
Habitat model response graphs. Partial conditional response graph of the relationship between the relative activity (number of passes) and habitat types included in the best-fitting negative binomial generalised linear models for *Mormopterus norfolkensis* and *Mormopterus* species 2. The grey shading represents 95% confidence intervals. Outliers are indicated by a number next to the point which specifies the value. FWW - freshwater wetland; DSF - dry sclerophyll forest and woodland; DSFS - dry sclerophyll forest and woodland on sand; UBUSH - urban bushland remnant; UMAT - urban matrix; URB - urban sites; MAN - mangrove forest; and SOFF - swamp oak forest.

Habitat type best predicted the activity levels of both *M. norfolkensis* and *M*. species 2 in the best-fitting GLMs compared to other environmental variables sampled. *Mormopterus norfolkensis* was more likely to be active in freshwater wetland, swamp oak forest and dry sclerophyll forest on sand compared to dry sclerophyll forest (P < 0.001), in the best fitting model (Table 1; Figure 4). Urban habitats were less likely to be used by *M. norfolkensis* than dry sclerophyll forest in the best-fitting model, but this was not significantly different (Table 1; Figure 4). There were two other models with strong support (within 2 AICc points of the best-fitting model) and four models in the 95% confidence set for *M. norfolkensis*. In the first supported model, *M. norfolkensis* was likely to be more active at low elevations and in areas without urban land-use within 500 m (Table 1). In the second supported model, *M. norfolkensis* was likely to be more active in areas with no urban land-use within 500 m and that had sandplain and floodplain soils compared to soils on undulating slopes (Table 1). ROC plots indicated that the best-fitting model was an excellent fit to the data (AUC = 0.92; Table 1) and explained a considerable portion of the variation in the data (Nagelkerke’s R^2^ = 0.78). Other models were poorer fitting (AUC < 0.85) and explained less variation in the data (Nagelkerke’s R^2^ < 0.70; Table 1).

**Table 1:** Summary from habitat analysis of a) *Mormopterus norfolkensis* and b) *M*. species 2 surrounding a large *M. norfolkensis* maternity roost.

Variable (s)	Estimate	Standard Error	Z	p	Log(L)	AICc	Wi	▵AICc	ROC	R^2^
*a) Mormopterus norfolkensis*										
**Model 1 - Best-fitting model**					–89.17	192.89	0.38	0	0.92	0.76
Intercept	–0.98	0.76	–1.29	0.20						
DSF versus FWW	3.75	0.90	4.18	<0.001*						
DSF versus SOFF	3.62	0.99	3.65	<0.001*						
DSF versus DSFS	3.66	0.91	4.01	<0.001*						
DSF versus URB	–1.32	1.33	–0.99	0.322						
**Model 2**					–92.19	193.53	0.19	0.64	0.83	0.66
Intercept	–0.65	0.70	–0.93	0.353						
Low elevation	3.41	0.74	4.58	<0.001*						
Urban land-use presence within 500 m	–1.75	0.65	–2.69	0.007*						
**Model 3**					–91.26	194.29	0.18	1.41	0.85	0.70
Intercept	–0.70	0.69	–1.02	0.307						
Urban land-use presence within 500 m	–1.69	0.67	–2.53	0.011*						
Undulating Slope versus Sandplain	3.29	0.82	4.00	<0.001*						
Undulating Slope versus Floodplain	3.60	0.77	4.66	<0.001*						
*b) Mormopterus species 2*					–101.76	221.01	0.93	0	0.76	0.77
Intercept	0.00	0.52	0	1.000						
DSF versus FWW	3.16	0.63	4.99	<0.001*						
DSF versus SOFF	0.88	0.76	1.15	0.251						
DSF versus DSFS	2.42	0.65	3.72	<0.001*						
DSF versus UBUSH	1.03	0.76	1.36	0.173						
DSF versus UMAT	–0.92	1.00	–0.92	0.359						

* denotes significant coefficients at α = 0.05.

FWW - freshwater wetland; DSF - dry sclerophyll forest and woodland; DSFS - dry sclerophyll forest and woodland on sand; UBUSH - urban bushland remnant; UMAT - urban matrix; MAN - mangrove forest; and SOFF - swamp oak forest.

Total activity was modelled using generalised linear models with a negative binomial distribution. Data presented includes the coefficient estimates, Nagelkerke’s R^2^ and ROC AUC values for the best-fitting and supported (within 2 AICc) models.

*Mormopterus* species 2 was more likely to occur in freshwater wetland and dry sclerophyll forest on sand compared to dry sclerophyll forest (P < 0.001; Table 1; Figure 4), in the best fitting model. There was no significant difference in the activity of *M.* species 2 in swamp oak forest, urban bushland and urban matrix compared to dry sclerophyll forest habitat types (Table 1; Figure 4). However, there was a trend for *M*. species 2 to be more active in swamp oak forest and urban bushland and less active in the urban matrix than the dry sclerophyll forest (Table 1; Figure 4). There was one other model in the 95% confidence set for *M.* species 2, but it did not have strong support. ROC plots indicated that the best-fitting model was a fair fit to the data (AUC = 0.76) and it explained considerable variation in the data (Nagelkerke’s R^2^ = 0.77; Table 1). There was no evidence of spatial autocorrelation in the residuals for models in the 95% confidence set for either of the study species (P > 0.564).

### Validation of regional-scale habitat models

**Figure 5:**
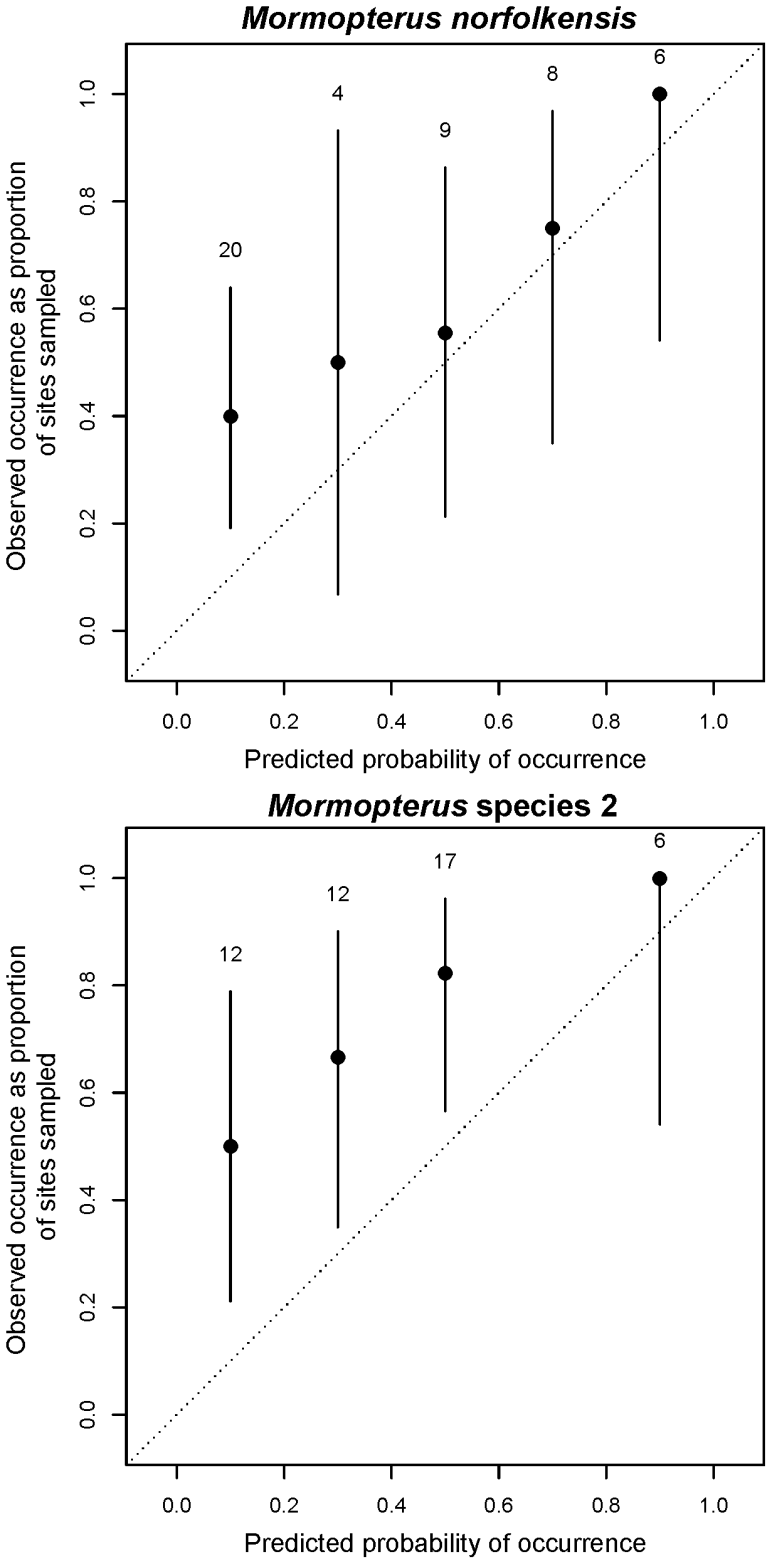
Regional model calibration plots. Calibration plots of the proportion of observed occurrence (collected during this study), against the predicted values of regional-scale probability of occurrence mapping [25] with 95% confidence intervals, for *Mormopterus norfolkensis* and *Mormopterus* species 2. Sample size in each bin is given by the numbers above. A close fit to the diagonal indicates good model calibration.

**Figure 6:**
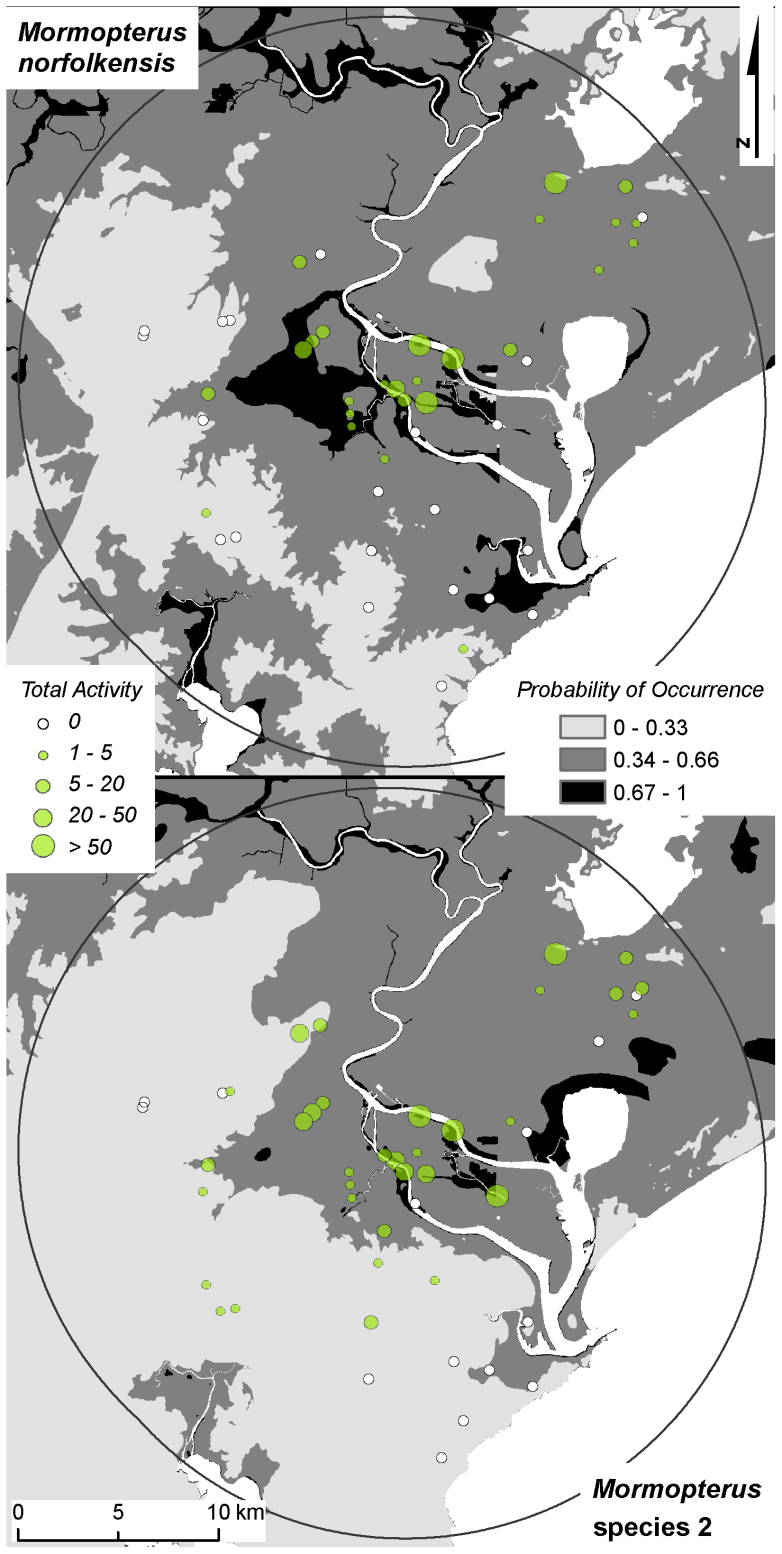
Spatial visualisation of prediction errors. Regional-scale probability of occurrence maps [25] presented at the local-scale of the study for *Mormopterus norfolkensis* and *Mormopterus* species 2 with activity levels from the local study overlaid. The probability of occurrence of each species from the regional-scale models was classified into three categories to aid visualisation of prediction errors. The sensitivity  =  specificity occurrence threshold (0.34) obtained from the local-scale study was used to define the lowest category.

Regional-scale model accuracy measures were calculated using a probability of occurrence threshold (above which we classify a presence and below which we classify an absence) of 0.34 for both species, as determined from the value of the intersection of sensitivity and specificity measures (see Figure S1). The regional-scale models had a good fit to the local data for both species as measured by AUC (> 0.72; Table 2), while kappa and TSS accuracy measures were slight to fair (0.255 – 0.317; Table 2). Presence records occurred generally proportional to predictions, indicating a good level of model calibration for *M. norfolkensis* (Figure 5). *Mormopterus norfolkensis* records did tend to occur more often than predicted in areas mapped as having a low probability of occurrence (0 – 0.4 probability of occurrence; Figure 5). However, prevalence was not greatly under-estimated for *M. norfolkensis* overall (Figure 5). Figure 6 illustrates the predictive accuracy of the regional-scale mapping for *M. norfolkensis*, displaying false negatives in the north of the study area (which coincides with the dry sclerophyll forest on sand habitat type) and false positives in the low-lying parts of Newcastle city. *Mormopterus* species 2 was more prevalent than predicted by regional-scale models (Figure 5) and false negatives occurred in western parts of the study area, coinciding with dry sclerophyll forest habitat types (Figure 6).

**Table 2:** Predictive accuracy of regional-scale models [25] to local-scale data collected during this study.

Accuracy measure	*Mormopterus norfolkensis*	*Mormopterus* species 2
AUC	0.757	0.719
Kappa	0.313	0.255
TSS	0.317	0.292
CCR	0.660	0.660
Sensitivity	0.667	0.676
Specificity	0.65	0.615
Prevalence	0.574	0.723

AUC – area under the curve calculated from the receiver operating characteristic; TSS – true skill statistic; CCR – correct classification rate.

The confusion matrix was constructed using a probability threshold of 0.34 for both species as determined by the intersection of sensitivity and specificity values.

## Discussion

Few studies test the performance of habitat models against systematically collected and independent datasets [54]–[56]. Our findings suggest that whilst the regional-scale habitat models are useful to describe habitat for these poorly understood species, they should be used cautiously at a local-scale, particularly when the consequences of errors are severe. Habitat type categories best predicted the activity of both species, compared to other environmental variables sampled, confirming previous findings that both species are more active in floodplains and that the threatened *M. norfolkensis* avoids urban areas. Additionally, these habitat type categories are easily communicated to land managers, illustrating that they are not only good summaries of environmental variation at a local-scale, but also practical tools for conservation.

### Local-scale habitat models

Both species were recorded more frequently and site activity levels were higher than recorded during previous studies in both urban and forested areas [14], [16], [57], [58]. Activity levels were also considerably higher than those recorded for the regional-scale study, where mangroves were not sampled and freshwater wetlands were under-sampled [25]. This allowed habitat use to be modelled using activity levels, which are preferable to presence/absence data that may inflate the importance of some habitats for mobile insectivorous bats due to false positives. The activity of both *M. norfolkensis* and *M*. species 2 was best explained by habitat type categories, which were based on vegetation communities that reflect environmental conditions such as soil type, climate, geology and topography. Habitat types may also incorporate other aspects such as vegetation structure, condition, disturbance history and the presence of important components such as hollow-bearing trees or rock outcrops, depending on the target species and the project aims. In this way, habitat types are likely to better represent the environmental variation in a particular area, compared to the combination of a small number of raw variables (such as elevation, aspect, etc). The classification of landscapes into different habitat types by experts is difficult at large spatial scales and has been previously found to offer little benefit over raw environmental variables [59]. However, we found that the creation of habitat type spatial layers can be efficiently compiled based on vegetation mapping combined with local expert knowledge for small study areas. Additionally, broad habitat type categories are easily communicated to conservation managers and authorities, which is beneficial compared to interpreting complex relationships with multiple raw variables.

*Mormopterus norfolkensis* and *M*. species 2 were more active in mangrove forest than any other habitat type sampled. This is most likely due to the abundant hollow resources in the Hunter Estuary mangrove forests, which have been found to be important roosting habitat for *M. norfolkensis* maternity colonies [30]. The percentage foraging activity in the mangrove forest was relatively low for *M. norfolkensis* and radio-tracking of bats indicated that they quickly move out of mangrove forests upon exiting roosts [30]. Additionally, very little *M. norfolkensis* activity has been recorded near hollow-depauperate mangrove forests elsewhere in NSW [60], [61]. As such, we suggest that mangrove forests within the study area represent roosting habitat for *M. norfolkensis* and that the foraging activity recorded during this study was opportunistic as bats exited and returned to roosts. Mangrove forests may not be as important for *M*. species 2 with less activity recorded and almost 45 times fewer *M*. species 2 individuals were captured during trapping within the Hunter Estuary mangrove forests [30].

When the mangrove forest sites were excluded from analyses, we found that both *M. norfolkensis* and *M*. species 2 were more active in freshwater wetland habitats and percentage foraging activity was also greatest in freshwater wetland for both species. Riparian habitats often support high levels of bat activity, which is likely to be due to high insect prey abundance [62]–[66]. Additionally, more productive soils (as are often associated with floodplains) have also been found to be related to high bat activity levels [14], [67]. These productive low-lying floodplain areas were previously predicted to be high habitat quality by regional habitat mapping for *M. norfolkensis* and *M*. species 2 [25], [68] and our study confirms the importance of these habitats.

Both *M. norfolkensis* and *M*. species 2 were more active in dry sclerophyll forest on sand compared to the dry sclerophyll forest on low undulating hills. The Tomago sandbeds (where this habitat type occurs) have characteristics (sandy soil and low vegetation height) that are typically considered to be associated with nutrient-poor environments. This contrasts with the high activity of both *M. norfolkensis* and *M*. species 2 recorded in productive floodplain areas of the study area. It is possible that the high activity is attributed to the presence of hollow-bearing trees that provide roosting habitat for these species. However, koalas (*Phascolarctos cinereus*) which have been linked to high fertility and clay soils in Victoria and Queensland [69], [70], also prefer these sandy soils within the study area [70], [71]. This occurrence of preferred koala habitat on the sandbeds has been attributed to the historical clearing of more suitable floodplain habitats [70], [72]. However, it is possible that the Tomago sandbeds are actually more productive than the sandy soil suggests, perhaps due to the high water table. Further investigation into the factors that drive productivity, particularly in relation to ground water would be valuable.

### Differential habitat use

Whilst floodplain habitats were used by both morphologically similar species, there were also differences in habitat use between the two species. Most notably, *M. norfolkensis* activity was negatively associated with urban areas, whereas urban bushland was positively associated with *M*. species 2 activity levels. *Mormopterus norfolkensis* was never recorded in the urban matrix and rarely in urban bushland remnants and this confirms previous assertions that *M. norfolkensis* avoids urban habitats [14], [25]. Habitat mapping from regional-scale models indicated that urban habitats were only marginal habitat for *M*. species 2 [25]. This is also consistent with our local study where *M*. species 2 was recorded at only one urban matrix site and whilst it was recorded relatively frequently in urban bushland, activity was low and foraging was rarely recorded. *Mormopterus norfolkensis* may be particularly sensitive to disturbance associated with urbanisation such as noise and artificial lighting (in contrast with *M*. species 2 which has been found to be positively associated with urban lighting [73]), but this requires explicit testing. Alternatively, these subtle differences in habitat use by *M. norfolkensis* and *M*. species 2 may represent spatial segregation to either prevent competitive interactions, or in response to them [74], [75]. Interspecific interactions are poorly understood for bat communities and further research into competition and niche partitioning is required.

### Validation of regional-scale habitat models

The descriptions of habitat use from regional-scale models were consistent with those from local-scale models. Additionally, the discrimination ability of regional-scale mapping, as assessed by AUC was fair to good. However, threshold-based accuracy measures (kappa and TSS) indicated that the regional models performed poorly with both false positive and false negative occurrences for *M. norfolkensis* and *M*. species 2 at a local-scale. Additionally, model calibration for *M*. species 2 suggested a systematic bias in which the regional-scale model underestimates species prevalence [12]. The broad level of agreement between these models suggests that they are useful to describe general habitat for these poorly understood species. However, our findings also illustrate that regional-scale models should not be used without testing for fine-scale prediction of habitat use where high levels of certainty are required. This is particularly the case when the consequences of errors are severe, such as using regional-scale models to allocate land for development or conservation.

### Management implications

The high level of use of floodplain areas and the avoidance of urban areas by *M. norfolkensis* suggest that its threatened conservation status is warranted. Floodplains are subject to high anthropogenic pressures from agriculture and urban development, with less than 30% of floodplain vegetation communities remaining in NSW [76]. Whilst wetland restoration has been found to have a positive effect on local bat communities elsewhere [77], it is important to monitor how particular management actions such as draining, insect spraying and installation of tidal floodgates affect insect and bat communities and these factors would benefit from further investigation. The findings of both regional and local-scale habitat models suggest that conservation efforts should focus on conserving and restoring floodplains, the sensitive management of riparian areas on private land and limiting urbanisation in these areas.

## Supporting Information

Figure S1 Sensitivity and probability of occurrence threshold. Effect of the probability of occurrence threshold on the sensitivity (dotted line), specificity (dashed line) and kappa (black line) accuracy measures for regional-scale models [25] tested against independent local-scale data collected for *Mormopterus norfolkensis* and *Mormopterus* species 2.
(TIF)

Table S1 Description of candidate variables used in the analyses. Values are mean ± se (range) or frequency of ordinal values. A patch was defined as > 10 trees occurring together and patch sizes > 500 m^2^ were mapped.
(DOCX)
