# Correction: Are Regional Habitat Models Useful at a Local-Scale? A Case Study of Threatened and Common Insectivorous Bats in South-Eastern Australia

**DOI:** 10.1371/journal.pone.0089702

**Published:** 2014-02-26

**Authors:** The PLOS ONE Staff

Figure 6 is incorrect. The authors have provided a corrected version here.

**Figure 6:**
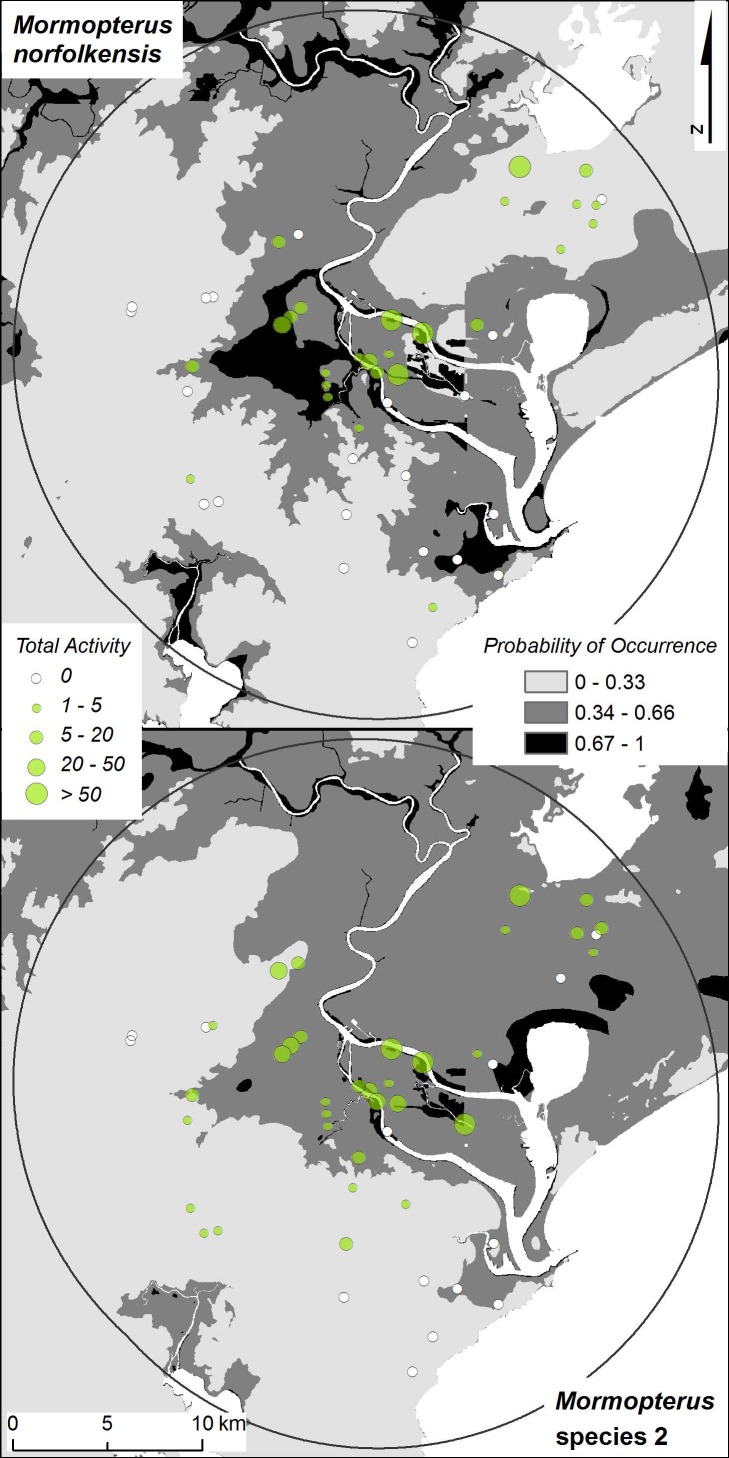
Spatial visualisation of prediction errors. Regional-scale probability of occurrence maps [25] presented at the local-scale of the study for Mormopterus norfolkensis and Mormopterus species 2 with activity levels from the local study overlaid. The probability of occurrence of each species from the regional-scale models was classified into three categories to aid visualisation of prediction errors. The sensitivity  =  specificity occurrence threshold (0.34) obtained from the local-scale study was used to define the lowest category.
