# Supplementary material for: Are Regional Habitat Models Useful at a Local-Scale? A Case Study of Threatened and Common Insectivorous Bats in South-Eastern Australia
Source: PLoS One. 2013 Aug 19;8(8):e72420. doi: 10.1371/journal.pone.0072420 (PMC3747113; doi:10.1371/journal.pone.0072420)
Supplement: Table S1 — Description of candidate variables used in the analyses. Values are mean ± se (range) or frequency of ordinal values. A patch was defined as > 10 trees occurring together and patch sizes > 500 m2 were mapped. (DOCX) [file pone.0072420.s002.docx]

Supplementary Material - Table S1 - Candidate Variables

Table S1: Description of candidate variables used in the analyses. Values are mean ± se (range) or frequency of ordinal values. A patch was defined as > 10 trees occurring together and patch sizes > 500 m^2^ were mapped.

| **Candidate Variable** | **Scale** | **Description / source** | **Source** | **Values (n = 47)** |
| --- | --- | --- | --- | --- |
| HabType | Site | Original stratification unit: freshwater wetland (FWW); dry sclerophyll forest and woodland (DSF); dry sclerophyll forest and woodland on sand (DSFS); urban bushland remnant (UBUSH); urban matrix (UMAT); mangroves (MAN) and swamp oak forest (SOFF). | Vegetation layer, aerial photography, site field data | FWW = 9; DSF = 8; DSFS = 8; SOFF = 5; UBUSH = 5; UMAT = 5; MAN = 7; URB_†_ = UMAT + UBUSH |
| Elev_low | Site | Elevation (m) converted to binary elevation categories for analyses (1 is <= 10; 0 is > 10) | DEM | 19.0 ± 3.6 (0 – 95.2) |
| Slope | Site | Slope (degrees). Excluded from analyses due to little variation | DEM | 1.7 ± 0.4 (0 – 15) |
| NearHBT | Site | Nearest hollow-bearing tree (m) | Field data | 111.5 ± 26.0 (0 – 750) |
| NearPatch | Site | Distance to nearest woody vegetation patch (m) | Vegetation layer | 149.7 ± 48.7 (0 – 1441.7) |
| NearUrb_close | Site | Distance to nearest urban land-use (m) converted to binary category where 1 is ≤ 500 m and 0 is > 500 m. | Urban land-use layer | 0 = 23  1 = 24 |
| NearRiv | Site | Distance to nearest major river (m). Log transformed for analysis | Aerial photography | 2812.0 ± 377.2 (60.7 – 9082) |
| DistMang | Site | Distance to mangrove maternity roost patches (m) | GIS analysis | 6256.4 ± 570.0  (0 – 12745.2) |
| Soil | Site | Broad soil landscape type adapted from [[23](#_ENREF_23)]. Categorical variable: undulating slope; floodplain; or sandplain | Soil landscapes [[23](#_ENREF_23)] | Floodplain = 22; Sandplain = 10; Undslope = 15 |
| FlyWidth_Narrow | Site | Binary flyway width category, where 1 is ≤ 10 m. A flyway is an opening where vegetation is < 1m in height. | Site field data | 0 = 25  1 = 22 |
| %Veg_cat | Landscape (0.25, 0.5, 1, 2.5, 5km buffers) | Percent vegetation within buffer of sample site converted to ordinal categories for analyses were 1 is ≤ 10 %; 2 is ≤ 80 % and 3 is > 80 % | Vegetation layer | 0.25: 1 = 14; 2 = 13; 3 = 20  0.5: 1 = 14; 2 = 19; 3 = 14  1: 1 = 13; 2 = 27; 3 = 7  2.5: 1 = 13; 2 = 34; 3 = 0  5: 1 = 1; 2 = 46; 3 = 0 |
| PatDens_low | Landscape (0.25, 0.5, 1, 2.5, 5km buffers) | Patch density – number of patches per 100 ha within the buffer of sample site. Converted to a binary variable where 1 is ≤ 5 and 0 is > 5 | GIS analysis - vegetation layer | 0.25: 0 = 39; 1 = 8  0.5: 0 = 20; 1 = 27  1: 0 = 17; 1 = 30  2.5: 0 = 13; 1 = 34  5: 0 = 18; 1 = 29 |
| MaxPat_cat | Landscape (0.25, 0.5, 1, 2.5, 5km buffers) | Maximum remnant vegetation patch size (ha) within buffer of sample site. Converted to ordinal categories where 1 is ≤ 100 ha; 2 is ≤ 500 ha and 3 is > 500 ha. | GIS analysis - vegetation layer | 0.25: 1 = 26; 2 = 15; 3 = 6  0.5: 1 = 23; 2 = 15; 3 = 9  1: 1 = 20; 2 = 16; 3 = 11  2.5: 1 = 11; 2 = 15; 3 = 21  5: 1 = 0; 2 = 12; 3 = 35 |
| MinPat_low | Landscape (0.25, 0.5, 1, 2.5, 5km buffers) | Minimum remnant vegetation patch size (ha) within buffer of sample site. Converted to binary variable where 1 is ≤ 1 ha; 0 is > 1 ha. | GIS analysis - vegetation layer | 0.25: 0 = 26; 1 = 21  0.5: 0 = 21; 1 = 26  1: 0 = 5; 1 = 42  2.5: 0 = 0; 1 = 47  5: 0 = 0; 1 = 47 |
| AWMPat_cat | Landscape (0.25, 0.5, 1, 2.5, 5km buffers) | Area weighted mean remnant vegetation patch size (ha) within buffer of sample site. Converted to ordinal categories where 1 is ≤ 50 ha; 2 is ≤ 250 ha and 3 is > 250 ha. | GIS analysis - vegetation layer | 0.25: 1 = 25; 2 = 10; 3 = 12  0.5: 1 = 23; 2 = 8; 3 = 16  1: 1 = 20; 2 = 11; 3 = 16  2.5: 1 = 14; 2 = 17; 3 = 16  5: 1 = 0; 2 = 23; 3 = 24 |
| EdgDens_cat | Landscape (0.25, 0.5, 1, 2.5, 5km buffers) | Density of edge habitat (km / 100 ha) within buffer of sample site. Derived from patch perimeters – does not include scattered trees. Converted to ordinal categories where 1 is ≤ 1 km per 100 ha; 2 is ≤ 5 km per 100 ha and 3 is > 5 km per 100 ha. | GIS analysis - vegetation layer | 0.25: 1 = 14; 2 = 13; 3 = 20  0.5: 1 = 13; 2 = 11; 3 = 23  1: 1 = 10; 2 = 12; 3 = 25  2.5: 1 = 4; 2 = 22; 3 = 21  5: 1 = 1; 2 = 29; 3 = 17 |
| %CoreAr_pres | Landscape (0.25, 0.5, 1, 2.5, 5km buffers) | Percent core area within buffer of sample site. Core area was calculated as the central portion of each remnant vegetation patch > 100 m from the edge. Converted to binary core area presence category where 1 is ≥ 5 %; 0 is < 5 %. | GIS analysis - vegetation layer | 0.25: 0 = 25; 1 = 22  0.5: 0 = 23; 1 = 24  1: 0 = 22; 1 = 25  2.5: 0 = 20; 1 = 27  5: 0 = 15; 1 = 32 |
| MeanCore_cat | Landscape (0.25, 0.5, 1, 2.5, 5km buffers) | Mean core area (ha) within buffer of sample site. Core area was calculated as the central portion of each remnant vegetation patch > 100 m from the edge. Converted to ordinal categories where 1 is 0 ha; 2 is ≤ 5 ha and 3 is > 5 ha. | GIS analysis - vegetation layer | 0.25: 1 = 21; 2 = 20; 3 = 6  0.5: 1 = 16; 2 = 26; 3 = 5  1: 1 = 13; 2 = 27; 3 = 7  2.5: 1 = 1; 2 = 41; 3 = 5  5: 1 = 0; 2 = 47; 3 = 0 |
| CoreDens_cat | Landscape (0.25, 0.5, 1, 2.5, 5km buffers) | Number of core areas of remnant vegetation patches per 100 ha within buffer of sample site. Converted to ordinal categories where 1 is ≤ 1 core area per 100 ha; 2 is ≤ 10 core areas per 100 ha and 3 is > 10 core areas per 100 ha. | GIS analysis - vegetation layer | 0.25: 1 = 21; 2 = 1; 3 = 25  0.5: 1 = 16; 2 = 5; 3 = 26  1: 1 = 14; 2 = 4; 3 = 29  2.5: 1 = 10; 2 = 17; 3 = 20  5: 1 = 1; 2 = 38; 3 = 8 |
| %CorePat_cat | Landscape (0.25, 0.5, 1, 5km buffers) | Average percent of a patch that is core area within buffer of sample site. Converted to ordinal categories where 1 is ≤ 10 %; 2 is ≤ 30 % and 3 is > 30 %. | GIS analysis - vegetation layer | 0.25: 1 = 24; 2 = 3; 3 = 20  0.5: 1 = 22; 2 = 4; 3 = 21  1: 1 = 15; 2 = 12; 3 = 20  2.5: 1 = 6; 2 = 27; 3 = 14  5: 1 = 0; 2 = 44; 3 = 3 |
| %FWW_pres | Landscape (0.25, 0.5, 1, 2.5, 5km buffers) | Percent of freshwater wetland within buffer of sample site. Converted to a binary presence category where 1 is > 5 % and 0 is ≤ 5 %. | GIS analysis - vegetation layer | 0.25: 0 = 37; 1 = 10  0.5: 0 = 36; 1 = 11  1: 0 = 33; 1 = 14  2.5: 0 = 30; 1 = 17  5: 0 = 23; 1 = 24 |
| %URB_pres | Landscape (0.25, 0.5, 1, 2.5, 5km buffers) | Percent of urban land-use within buffer of sample site. Converted to a binary presence category where 1 is > 5 % and 0 is ≤ 5 %. | GIS analysis - vegetation layer | 0.25: 0 = 37; 1 = 10  0.5: 0 = 30; 1 = 17  1: 0 = 22; 1 = 25  2.5: 0 = 11; 1 = 36  5: 0 = 2; 1 = 45 |
| AvgMinTemp | Site | Average minimum nightly temperature (°C) recorded during the sampling period of each site. | nearest BOM station | 19.3 ± 0.3 (15.3 – 23.1) |
| AvgAnnRain | Site | Average annual rainfall (mm) | GIS – average annual rainfall | 1079.8 ± 6.5  (1009 – 1182) |

_†_ The UBUSH and UMAT habitat type categories were combined to form the URB category for the *M. norfolkensis* models as no *M. norfolkensis* activity was recorded at any UMAT site.
